# Dietary fiber intake and mortality among survivors of liver cirrhosis: A prospective cohort study

**DOI:** 10.1016/j.heliyon.2023.e16170

**Published:** 2023-05-09

**Authors:** Zahra Hariri, Azita Hekmatdoost, Fereshteh Pashayee-khamene, Sara Karimi, Salehe Ahmadzadeh, Zahra Yari

**Affiliations:** a Clinical Nutrition and Dietetics Department, Faculty of Nutrition Sciences and Food Technology, National Nutrition and Food Technology Research Institute, Shahid Beheshti University of Medical Sciences Tehran, Tehran, Iran; b Department of Nutrition Research, National Nutrition and Food Technology Research Institute and Faculty of Nutrition Sciences and Food Technology, Shahid Beheshti University of Medical Sciences Tehran, Tehran, Iran

**Keywords:** Cirrhosis, Mortality, Soluble fiber, Insoluble fiber, Cohort study

## Abstract

**Background:**

Liver cirrhosis is associated with significant nutritional risks and poor survival rates. Little is known about the impact of dietary factors on metabolic complications and mortality from cirrhosis.

**Aim:**

The present study investigated the potential associations between dietary fibers and the risk of cirrhosis-related mortality.

**Methods:**

In this prospective study, 121 ambulatory cirrhotic patients with more than six months of cirrhosis diagnosis were followed-up for 4 years. Dietary intakes were assessed using a 168-item semi-quantitative validated food frequency questionnaire. Crude and multivariable-adjusted hazard ratios (HRs) and 95% confidence intervals (CIs) were estimated through cox proportional hazards regression models.

**Results:**

Comparing the highest versus the lowest tertile, soluble and insoluble fiber intake was associated with 62% (HR = 0.38, 95% CI = 0.045–3.5, p trend = 0.047) and 73% (HR = 0.27, 95% CI = 0.06–1.2, p trend = 0.021) lower mortality risk, respectively, after full adjustment for potential confounders. Higher intakes of total fiber were inversely but non-significantly associated with mortality risk.

**Conclusion:**

Comprehensive assessment of dietary fiber intake associations with cirrhosis-related mortality showed that higher intakes of soluble and insoluble fiber were significantly associated with reduced mortality risk.

## 1 Introduction

Liver cirrhosis is the final stage of chronic hepatic diseases, which is allied with a high rate of morbidity and mortality [1]. About two million deaths due to liver diseases are reported worldwide yearly, and more than one million of them are caused by liver cirrhosis [2,3]. The number of deaths, disability-adjusted life years and the proportion of all global deaths due to cirrhosis are increasing [4]. In Iran, the Global Burden of Disease (GBD) project in 2017 showed that 1.42% of all deaths were caused by cirrhosis and other liver diseases [5].

Diet plays a vital role in the etiology of liver cirrhosis and dietary modification is easy to implement with little risk of side effects or cost, so it can be offered to all patients [1]. It also has proven effects in managing non-alcoholic fatty liver disease (NAFLD), which is known as the most common cause of cirrhosis [6]. Dietary fiber is widely recognized as an essential part of a healthy diet and has been cited in previous studies which had assessed the effects of nutrition on chronic liver diseases [[7], [8], [9]]. Depending on solubility, dietary fiber can be divided into water-soluble and water-insoluble fiber [10]. Soluble fibers consist of gelforming substances, such as pectins, gums, some hemicelluloses, mucilages, and storage polysaccharides and insoluble fibers consist of structural and/or matrix fibers such as cellulose, part of hemicellulose and lignin [11,12]. The main sources of soluble fiber are fruits, vegetables and oats [11] and insoluble fiber is generally found in whole grains, brans and vegetables [13].

Accumulating evidence indicated that dietary fiber might decrease the risks of obesity, dyslipidemia and hyperglycemia, which are leading causes of non-alcoholic fatty liver disease and subsequently liver cirrhosis [14,15]. Moreover, dietary fiber could act as a prebiotic through fermentation in the colon, especially soluble fibers, and provide many other health benefits [16]. Dietary fibers also are known to improve insulin sensitivity and have anti-inflammatory properties [17,18].

According to previous studies, higher fiber intake is associated with lower mortality, particularly from cardiovascular, gastrointestinal, and inflammatory diseases [19,20]. However, little is known about the association of dietary fiber intake with cirrhosis-related mortality. In this study, we evaluated potential associations between dietary total, soluble and insoluble fibers with total mortality, using data from a prospective cohort study in cirrhotic patients.

## 2 Methods and materials

### 2.1 Study population

In total, 166 ambulatory cirrhotic patients with more than six months of cirrhosis diagnosis were recruited in this cohort study from 2016 to 2018 and were followed up to 30 April 2022, from two educational hospitals in Tehran, Iran including Ayatollah Taleqani Hospital and Shariati Hospital which are respectively affiliated to Shahid Beheshti University of Medical Sciences (SBMU), and Tehran University of Medical Sciences (TMUS). This cohort study was planned to follow up the participants for 4 years after enrollment. After entering the study, the patients were followed up annually. Participants received annual telephone calls during which follow-up questionnaires were completed regarding the occurrence of death or any medical event. At the end of 4 years, mortality and survival were determined.

**Fig. 1:**
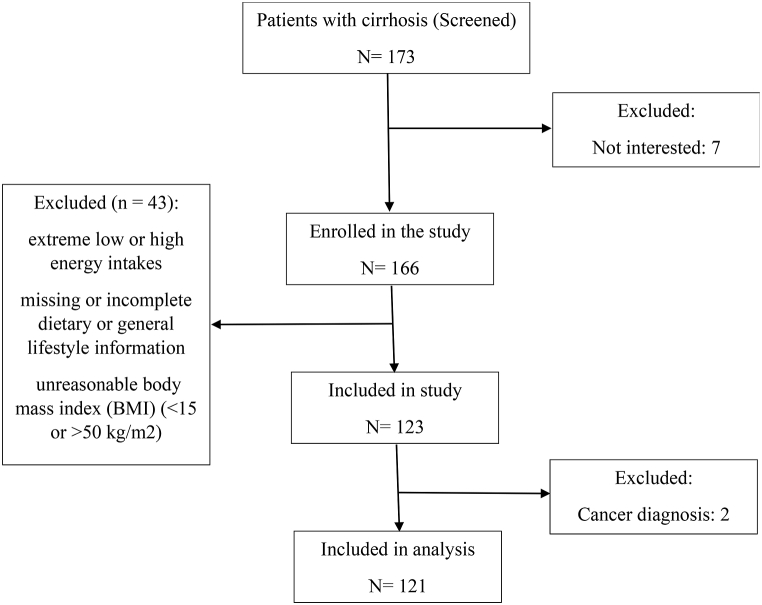
**Flowchart** of participant recruitment.

Exclusion criteria were: (1) being pregnant or lactating, (2) having diabetes mellitus, renal failure, chronic cardiac disease, malignancies, infectious disease, pancreatic insufficiency and acquired immune deficiency syndrome. We also excluded participants (n = 45) with extremely low or high energy intakes (<500 or >5000 kcal/day), diagnosis of cancer in the first year, missing or incomplete dietary or general lifestyle information, and those with an unreasonable body mass index (BMI) (<15 or >50 kg/m^2^). Protocol of this study was approved at National nutrition and Food Technology Research Institute (NNFTRI) ethics committee (Ir.sbmu.nnftri.1396.186). All participants provided written informed consent before enrollment. Finally, 121 participants (83 men and 38 women) were included in the analyses. The flow chart provides a description of the patients recruited and the sample sizes for analysis (Fig. 1).

### 2.2 Dietary assessment

At the time of enrollment, dietary intakes were collected through face-to-face interviews using a valid and reliable food frequency questionnaire (FFQ) with 168 items [21]. Typical portion sizes, frequency of consumption, and servings consumed each time for each food item were questioned. A daily, weekly or monthly intake of each food item was recorded and converted to grams using household measurements. The collected data were analyzed using Nutritionist IV software. The United States Department of Agriculture (USDA) food composition table (FCT) was used to calculate energy and nutrient contents. For traditional Iranian foods that were not provided by the USDA FCT, the Iranian food composition table was used. In addition to total fiber, the contents of insoluble and soluble fiber were calculated and expressed as grams per day.

### 2.3 Potential confounders

Basic characteristic data including age, sex, smoking and alcohol consumption, subjective global assessment tool (SGA), body mass index (BMI), etiology of cirrhosis, Model for end-stage liver disease (MELD) and Child-Pugh score were collected at the enrollment. In addition, each patient was weighed with minimal clothes, using a digital scale to the nearest 0.5 kg and height was measured without shoes, using a portable stadiometer to the nearest of 0.1 cm. Moreover, BMI was calculated by dividing the weight (in kilograms) by the square of the height (in meters). The nutritional status of each patient was estimated using the SGA based on Destky et al. study [22]. Based on this assessment, patients were divided into three categories: A: well-nourished, B: moderately malnourished and C: severely malnourished.

MELD and Child-Pugh scores were used to evaluate the prognosis and severity of liver cirrhosis. The MELD score was calculated by formula 9.57 × Ln (creatinine, mg/dl) + 3.78 × Ln (total bilirubin, mg/dl) + 11.2 × Ln (INR) + 6.43 [23]. Child-Pugh score was calculated which included the presence of ascites, encephalopathy, albumin, prothrombin time and serum bilirubin and patients were classified into three categories of A, B and C.

### 2.4 Statistical analysis

Participants were divided into three groups based on their dietary fiber intake. The basic characteristics of participants were compared among the tertiles of total dietary fiber using a one-way analysis of variance (ANOVA) test for continuous variables and the chi-squared (χ2) test for categorical variables. Multivariable-adjusted hazard ratios (HRs) and 95% confidence intervals (CIs) were estimated through cox proportional hazards regression models for all-cause mortality associated with the dietary total fiber, soluble fiber and insoluble fiber tertiles. Potential confounders, added in a following series: Model 1: adjusted for age (continuous), and sex (male, female); Model 2: additionally adjusted for energy intake (continuous), BMI (continuous), smoking (yes, no), and alcohol using (yes, no); and Model 3: additionally adjusted for etiology (virus, autoimmune, other), MELD (continuous) and Child-Pugh (A, B & C).

Potential interactions between major risk factors at baseline, including BMI, age, SGA, MELD and Child-Pugh and dietary intakes of total, soluble and insoluble fiber in relation to risk of mortality were tested using the likelihood ratio test (LRT). Person-years of follow-up was considered from the date of enrollment until the date of death, lost to follow-up or censoring on 30 April 2022, whichever occurred first. All the statistical analyses were performed using SPSS (version 19; SPSS Inc, Chicago, IL, USA) and significance level was set at α = 0.05.

## 3 Results

The mean age ± standard deviation (SD) of participants at baseline was 54.8 ± 11.9 years. Overall, 31.4% were women and the etiology of cirrhosis in 52.9% of patients was viral hepatitis. During 3955 person-month of follow-up, we documented 43 deaths (7 women, 36 men). Liver failure was responsible for 47% of deaths, cardiovascular diseases 40%, carcinoma 3% and other causes for 10% of deaths.

The mean of total calorie and daily fiber intake of patients was 1900 kcal and 15 g, respectively. The average intake of soluble and insoluble fiber was estimated to be about 8 and 7 g, respectively. The average BMI of the patients was estimated to be 27 kg/m^2^ and 37, 38 and 25% of them were normal, overweight and obese respectively. Also, alcohol consumption in 22% and smoking in 39% of patients were reported. The baseline characteristics of the participants according to the total dietary fiber tertiles are shown in Table 1. Viral hepatitis was found to be the main cause of liver cirrhosis in patients.

**Table 1:** Characteristics of participants according to the fiber intake tertile.

	Tertile of total fiber intake			
	T1	T2	T3	*P* value
Men, %	68.3	57.5	80	0.095
Age (y)	54 ± 12	55 ± 13	56 ± 9	0.735
Etiology of cirrhosis				0.028
Virus	52.5	54.1	62.2	
Autoimmune	40	37.8	13.5	
Other	7.5	8.1	24.3	
MELD score	12.4 ± 5	12.7 ± 5	11.4 ± 4	0.527
Child Pugh category (A/B/C)	%			0.566
A	60.7	70.6	73	
B, C	39.3	29.4	32.3	
Alcohol drinker	18.9	17.9	33.3	0.202
Smoker, %	44.7	32.5	43.6	0.473
Weight, kg	70.3 ± 17.8	72.7 ± 14.2	78.7 ± 16.3	0.058
Height, cm	164.7 ± 7.7	163.8 ± 8.7	167.5 ± 8.1	0.113
Body mass index, kg/m^2^	25.9 ± 5.6	27.3 ± 5	28.2 ± 5.1	0.149
Subjective global assessment				0.028
A	14.6	35	47.5	
B	65.9	50	45	
C	19.5	15	7.5	
Calorie intake (Kcal/day)	1718 ± 626	1956 ± 518	2140 ±‌ 601	0.049
Total fiber intake (g/day)	11.7 ± 3	16.8 ± 4	18.7 ± 4	<0.001
Insoluble fiber intake (g/day)	6.1 ± 3.1	9 ± 4.2	10.1 ± 3.6	<0.001
Soluble fiber intake (g/day)	5.5 ± 2.8	7.6 ± 3.5	8.8 ± 4.2	<0.001

Values are means ± SDs for continuous variables and percentages for categorical variables.

ANOVA for quantitative variables and χ^2^ test for qualitative variables.

Multivariable-adjusted hazard ratios (HRs) and 95% confidence intervals (CIs) for all-cause mortality associated with dietary fiber intake are shown in Table 2. Total fiber intake was inversely but non-significantly associated with risk of mortality. The risk of all-cause mortality was lower in patients in the last tertile (T3) of soluble fiber intake (HR = 0.38, 95% CI = 0.04–3.5, p trend = 0.047) in comparison with those in the first tertile (T1), after adjustment for all confounders. Similar results were achieved in the analysis of insoluble fiber. Higher intakes of insoluble fiber were associated with a lower risk of mortality in both model 2 (HR = 0.31, 95% CI = 0.07–1.4, p trend = 0.024) and model 3 (HR = 0.27, 95% CI = 0.06–1.2, p trend = 0.021). Dietary intakes of total, soluble and insoluble fiber were not significantly associated with mortality risk in the age- and sex-adjusted models.

**Table 2:** Hazard ratios for total mortality, according to the fiber intake tertile.

	Tertiles of fiber intake			*P* trend
Total fiber				
	T1	T2	T3	
No. of deaths	19	13	11	
Model 1	ref	0.42 (0.07–2.62)	0.52 (0.04–7.5)	0.264
Model 2	ref	0.64 (0.25–1.62)	0.41 (0.1–1.3)	0.123
Model 3	ref	0.67 (0.23–1.98)	0.37 (0.07–1.85)	0.071
**Soluble fiber**				
	T1	T2	T3	
No. of deaths	24	11	8	
Model 1	ref	0.81 (0.26–2.47)	0.5 (0.17–1.4)	0.089
Model 2	ref	0.49 (0.2–1.2)	0.32 (0.09–1.09)	0.060
Model 3	ref	0.69 (0.1–5.07)	0.38 (0.04–3.5)	0.047
**Insoluble fiber**				
	T1	T2	T3	
No. of deaths	22	12	9	
Model 1	ref	0.65 (0.11–3.92)	0.5 (0.2–1.3)	0.083
Model 2	ref	0.45 (0.16–1.23)	0.31 (0.07–1.4)	0.024
Model 3	ref	0.41 (0.16–1.05)	0.27 (0.06–1.2)	0.021

Cox proportional hazards regression models for estimating HRs and 95% CIs.

Model 1: adjusted for age and sex.

Model 2: additionally adjusted for energy intake, BMI, smoking and alcohol

Model 3: additionally adjusted for etiology, MELD and child.

**Fig. 2:**
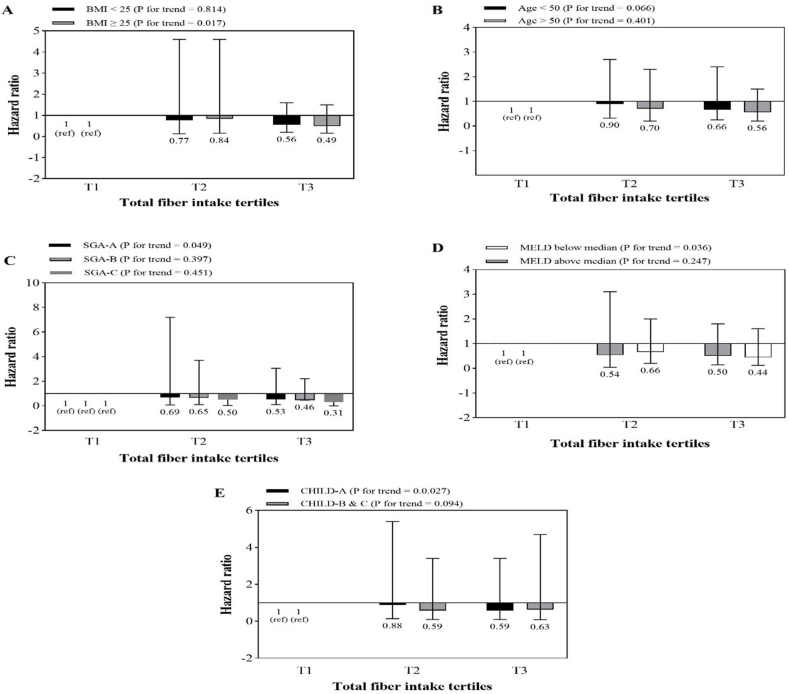
Multivariate hazard ratios of total fiber intake tertiles for cirrhosis-related mortality according to risk factor status at baseline (Cox proportional hazards regression models for estimating HRs and 95% CIs, multivariable models were adjusted for sex, age, energy intake, BMI, smoking, alcohol, etiology, MELD and child, except for the respective stratifying factor). Data are reported as HR (95% CI). **A**, BMI<25 vs ≥ 30 (P = 0.011 for interaction); **B**, age <50 years vs ≥ 50 years (P = 0.01 for interaction); **C**, SGA A vs B and C (P = 0.021 for interaction); **D**, MELD score below median vs above median (P < 0.001 for interaction); **E**, Child Pugh A vs B&C (P = 0.001 for interaction).

**Fig. 3:**
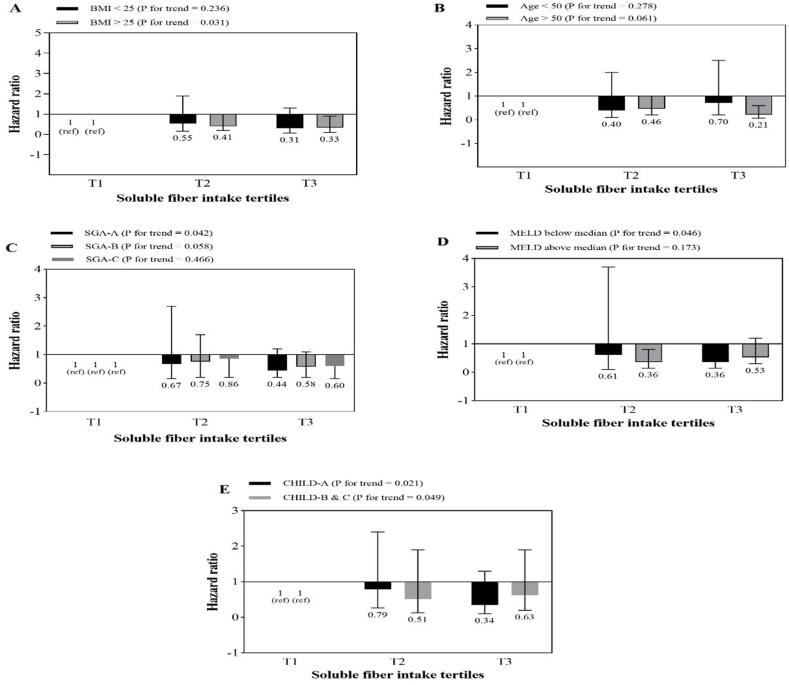
Multivariate hazard ratios of soluble fiber intake tertiles for cirrhosis-related mortality according to risk factor status at baseline (Cox proportional hazards regression models for estimating HRs and 95% CIs, multivariable models were adjusted for sex, age, energy intake, BMI, smoking, alcohol, etiology, MELD and child, except for the respective stratifying factor). Data are reported as HR (95% CI).

**Fig. 4:**
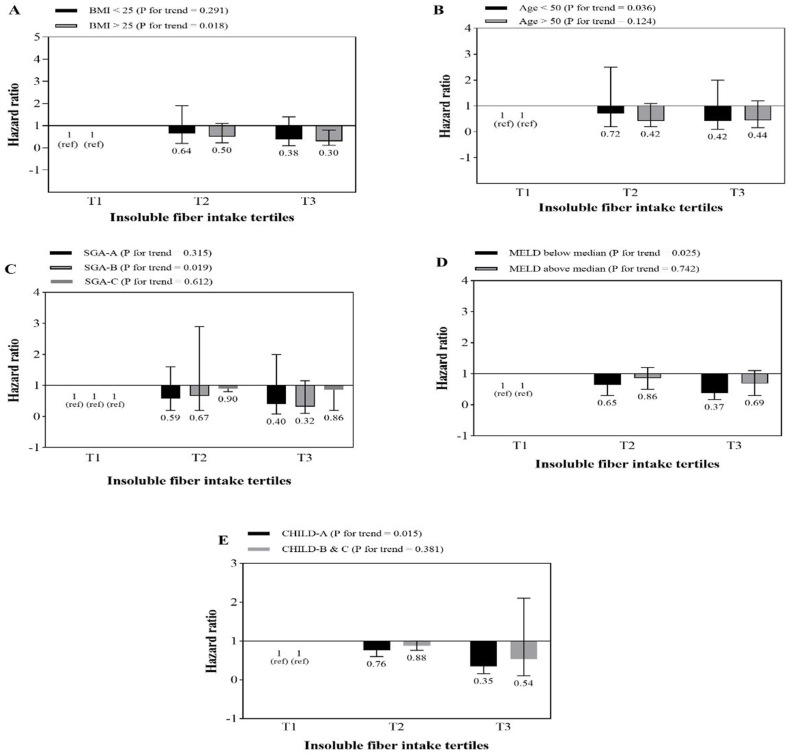
Multivariate hazard ratios of insoluble fiber intake tertiles for cirrhosis-related mortality according to risk factor status at baseline (Cox proportional hazards regression models for estimating HRs and 95% CIs, multivariable models were adjusted for sex, age, energy intake, BMI, smoking, alcohol, etiology, MELD and child, except for the respective stratifying factor). Data are reported as HR (95% CI).

The association between total fiber intake and mortality risk according to BMI, age, SGA, MELD and Child-Pugh stratification is shown in Fig. 2. A similar analysis for soluble and insoluble fiber are shown in Fig. 3, Fig. 4, respectively. The results showed that higher intakes of dietary fiber, including total, soluble and insoluble fiber, were significantly associated with a lower risk of total mortality in patients who were obese or overweight (BMI≥ 25), those with MELD scores above the median (>11), and those who were in the first category of Child-Pugh (A). Also, with an increase in insoluble fiber intake, the risk of mortality in patients under 50 compared to those over 50 years old showed a significant decrease (p = 0.036).

## 4 Discussion

To the best of our knowledge, the association of dietary total, soluble and insoluble fiber intake with the risk of mortality in cirrhotic patients has not been investigated yet. Comparing the highest versus the lowest tertile in the present cohort study showed that soluble and insoluble fiber intake was associated with 62% and 73% lower mortality risk, respectively, after full adjustment for potential confounders. Higher intakes of total fiber were inversely but non-significantly associated with the risk of mortality.

A meta-analysis study reported an inverse association between higher dietary fiber intake and all-cause mortality risk [24]. Similarly, the results of a recently published cohort study indicated an inverse association between fiber intake and the risk of mortality from chronic liver diseases, including cirrhosis [7]. The National Institutes of Health–American Association of Retired Persons Diet and Health Study cohort (993 deaths from chronic liver disease) reported an inverse association between dietary fiber intakes and chronic liver disease mortality (HR_Q5 vs. Q1_ = 0.37, 95% CI: 0.29–0.48) [7].

The biological mechanisms for the inverse associations of dietary fiber with cirrhosis remain to be fully elucidated. The imbalance of the intestinal microbiome has been proposed as one of the possible mechanisms in the pathophysiology of cirrhosis [25,26]. This effect might be attributed to the increased production of endotoxins or lipopolysaccharides by pathogenic bacteria (e.g., Enterobacteriaceae), which can impair intestinal integrity and exacerbate inflammation in the liver [27]. Dietary fibers, as prebiotics, can reduce liver oxidative stress and inflammation by improving intestinal dysbiosis and tight junction integrity. Prebiotics can normalize plasma endotoxin concentrations by stimulating the growth of Gram-positive bacteria such as Bifidobacteria and Lactobacillus and inhibiting the endotoxemic Gram-negative bacteria [28].

In addition, dietary fibers can also prevent cirrhosis by ameliorating NAFLD, one of the most important causes of cirrhosis. A 20-year multiethnic cohort with 2974 NAFLD cases and 29,474 matched controls reported an inverse significant association between total dietary fiber and risk of NAFLD (OR = 0.84; P = 0.003) [29]. In accordance, a large Chinese cross-sectional study showed that higher intakes of insoluble fiber were associated with a lower prevalence of newly-diagnosed NAFLD in men (OR = 0.60; P < 0.001) [30]. However, this study failed to show this association for soluble fiber. As a result of the fermentation of dietary fibers by gut bacteria, short-chain fatty acids (SCFA) are produced, which have been revealed to play a role in the pathogenesis of NAFLD [31]. It has also been demonstrated that SCFAs may suppress inflammation and lipid peroxidation [31]. Other potential mechanisms of dietary fibers that have been proposed in prevention and treatment of fatty liver are as follows: (1) reducing carbohydrate and fat absorption, (2) enhancing glycemic control (3) improving insulin sensitivity, (4) lipid-lowering effects and (5) reducing weight and adiposity [32,33]. The beneficial effects of dietary fiber in reducing encephalopathy in cirrhotic patients have also been reported in few studies [34,35]. This could be another explanation for the association of dietary fiber with reduced cirrhosis-related mortality. More investigation is needed to further clarify the potential mechanisms.

Investigating the association of fiber, separately by solubility, with the risk of cirrhosis-related mortality is one of the strengths of present cohort study. Prospective cohort design with 4-year follow-up in evaluating this association is another strength. Moreover, all potential confounders were considered in the analyses. Also, in this study, the relationship between total fiber intake and mortality risk was measured based on BMI, age, SGA, MELD and Child-Pugh classification. Our study also has some limitations. First, the study population was relatively small, limiting the precision of our effect estimates, therefore, results should be interpreted with caution, and confirmation from larger studies is warranted. Second, the use of FFQ is inevitably allied with recall bias and patients may over-report as well as underreport dietary intake. Third, about 15% of the enrolled patients were missing. Furthermore, as in many epidemiological studies, we cannot rule out that the results are partially biased by residual and unmeasured confounders and loss to follow-up. Conducting clinical trials in these patients to compare the difference in complications and mortality will add strength to this study.

## 5 Conclusion

In conclusion, we found significant inverse associations between soluble and insoluble dietary fiber intake with mortality in cirrhotic patients. Further studies are recommended to determine the effectiveness and the appropriate amount of fiber, including total, soluble and insoluble, in cirrhotic patients.

## Author contribution statement

Zahra Hariri: Conceived and designed the experiments; Wrote the paper.

Azita Hekmatdoost: Performed the experiments; Wrote the paper.

Fereshteh Pashayee-khamene: Contributed reagents, materials, analysis tools or data.

Sara Karimi, Salehe Ahmadzadeh: Contributed reagents, materials, analysis tools or data.

Zahra Yari: Conceived and designed the experiments; Performed the experiments; Analyzed and interpreted the data; Wrote the paper.

## Data availability statement

Data will be made available on request.

## Sources of support

No funding has been received for this study.

## Author declarations

The authors declare that they have no competing interests.

## Declaration of competing interest

The authors declare that they have no known competing financial interests or personal relationships that could have appeared to influence the work reported in this paper
